# Surgical treatment of a 36-year-old patient with asphyxiating thoracic dysplasia

**DOI:** 10.1093/icvts/ivab217

**Published:** 2021-08-03

**Authors:** Wenlin Wang

**Affiliations:** Department of Chest Wall Surgery, Guangdong Second Provincial People’s Hospital, Guangzhou, China

**Keywords:** Asphyxiating thoracic dysplasia, Operation, 36 years old

## Abstract

Asphyxiating thoracic dysplasia is a rare and dangerous genetic disease. Many children with this disease die early in life of severe hypoxia, and it is extremely rare that they survive to adulthood. We recently treated a 36-year-old patient who had asphyxiating thoracic dysplasia with a special surgical method and achieved satisfactory results. A review of the literature showed that this patient is the oldest surviving person with this condition.

## INTRODUCTION

Asphyxiating thoracic dysplasia (ATD), an extremely rare and dangerous disease, was first reported by Jeune in 1955 [1]. Most patients die before puberty, and few live to adulthood [2]. We recently treated a 36-year-old patient with ATD using a special surgical technique. After consulting the literature, we found that this patient is the oldest surviving person with this condition.

## PATIENT AND SURGICAL TECHNIQUE

**Figure 1:**
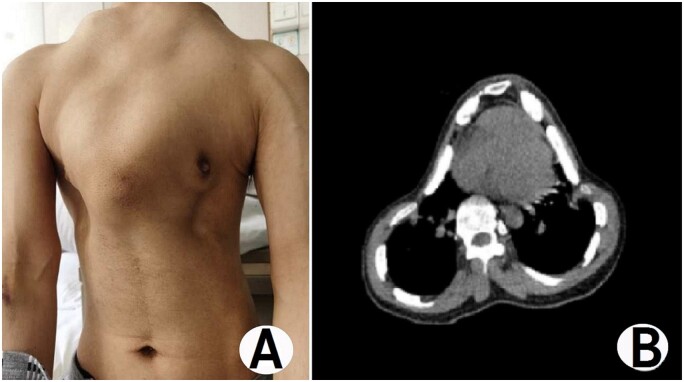
The appearance of the chest wall and computed tomography images of the patient. Preoperative chest wall appearance (**A**) and preoperative chest CT image (**B**).

**Figure 2:**
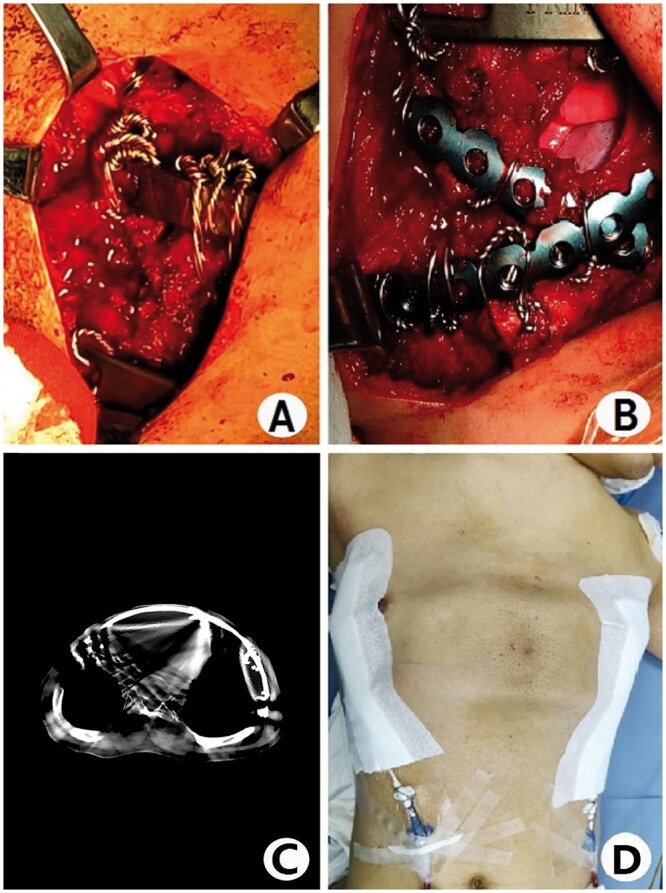
Perioperative and postoperative pictures. Operative pictures (**A** and **B**); postoperative chest computed tomography image (**C**); and postoperative appearance of the chest wall (**D**).

The patient was a 36-year-old man. He was admitted because of severe dyspnoea for 2 years and aggravation for 1 month. He was found to have a thoracic deformity after birth and was diagnosed with ATD at an early age. He often had dyspnoea after activities, but in the past 2 years, he had dyspnoea even at rest. One month before admission, he often felt that he was dying due to a lack of oxygen, so he came to our hospital for surgical treatment. He was short, only 145 cm tall. His chest wall narrowed (Fig. 1A). The anterior chest wall was convex, and the lateral walls were concave. He had an obvious spinal deformity. No obvious deformities were found in other parts of the body. An imaging examination showed an obvious thoracic deformity (Fig. 1B), obvious compression of the heart and lung, lordosis and scoliosis. His preoperative diagnosis was ATD. The operation was performed with the patient in a supine position. Incisions were made on both sides of the chest wall. Two tunnels were made in front of the sternum, and 2 arc-shaped steel bars were put into the tunnels. The ribs in the concave area were lifted and fixed to the bars with a number of steel wires around the ribs in the concave position (Fig. 2A). For the concave area in the lower part of the lateral chest wall, 2 short steel bars were placed above the concave area, and the structures in the concavity were also lifted and fixed to the bars (Fig. 2B). After the incisions were closed, the operation was completed. The chest wall deformity was significantly improved after the operation (Fig. 2C and D). His chest circumference was increased from 65 to 73 cm, and the oxygen saturation level was significantly improved from 91% to 96%. The endotracheal tube was removed immediately after the operation. His vital signs were stable on the first postoperative day, but mental symptoms began to appear on the second postoperative day. Because the blood gas analysis showed hypercapnia, he was intubated and placed on a ventilator immediately. On postoperative day 7, a tracheotomy was performed, and intermittent assisted breathing was provided. On postoperative day 23, his condition was stable and the assisted breathing was stopped. Two days later, the endotracheal tube was removed. In the early postoperative period, the patient felt obvious pain on the chest walls. We used an analgesia pump to relieve the pain, and the symptoms disappeared. He was discharged 1 week after the symptoms disappeared completely. An imaging examination before discharge showed that the deformity did not recur (Fig. 2C). He has been followed up for 5 months and has had no recurrence of anoxia.

## DISCUSSION

ATD can cause pathological changes in many parts of the body. The damage to the chest wall is the most obvious; it is manifested by the overall narrowing of the chest wall and local deformity. The narrowing of the chest wall can damage the respiratory function. Many children with this condition die of a lack of oxygen. The literature shows that few patients live to adulthood [2]. This patient was 36 years old and the oldest patient reported so far.

An operation to treat ATD was first performed in 1971 [3]. Several operations have been reported since then [4, 5]. Because this disease is extremely rare, the experience of each operation is limited. During our operation, with the help of steel bars, we not only corrected the median protrusion and bilateral concavity but also increased the capacity of the chest. Our method is different from those of all other authors. Although this patient developed respiratory failure at an early stage, which may be related to long-term chronic lung damage, the results still show that our method is effective.

## CONCLUSIONS

This man is the oldest patient with ATD reported in the literature. With our operation, we used special methods and achieved satisfactory results.

**Conflict of interest:** none declared.

## Reviewer information

Interactive CardioVascular and Thoracic Surgery thanks Larry R Kaiser and Nikolay O. Travin for their contribution to the peer review process of this article.
